# Revealing Topography Evolution of Glass Surface under Air Pollution by Atomic Force Microscope

**DOI:** 10.1155/2021/6650020

**Published:** 2021-04-10

**Authors:** Zhou Huaicheng, Wu Lei, Yu Bingjun, Qin Na

**Affiliations:** School of Mechanical Engineering, Southwest Jiaotong University, Chengdu 610031, China

## Abstract

Air pollution has become a matter of close concern to people with the continuous development of human society. However, the knowledge of air pollution mechanisms remains insufficient, and there is a lack of evaluation methods for actual pollution. In this paper, air pollution in Internet cafe was studied by detecting surface topography of glass slides after different exposure time by an atomic force microscope (AFM). It is found that the adsorption of air pollutants on glass surface undergoes initial aggregation, particle growth and interparticle deposition, and final full coverage. The chemical composition of contaminated glass surface was further analyzed by an X-ray photoelectron spectrometer, showing that the pollution was mainly composed of hydrocarbons regardless of exposure time. Cleaning experiments show that NaHCO_3_ solution can be the most effective one with saponification reaction and hydrolysis to remove the adsorbed contaminations. This study provides an alternative way for investigating air pollution and a reference for cleaning polluted material surfaces.

## 1. Introduction

As a hot issue today, air pollution is manifested as compound pollution resulting mainly from the discharge of soot and petroleum due to the rapid industrialization and urbanization and can be divided into indoor and outdoor air pollution. Outdoor air pollution, often called air pollution, is primarily caused by industrial waste gas and traffic exhaust. Studies have shown that air pollutants, such as sulfur dioxide, nitrogen oxides, and major particles, are the major contributors to the formation of PM2.5 [1, 2]. South Korean researchers surveyed 249 administrative units on the relationship between long-term exposure to air pollutants and cardiopulmonary mortality. The data showed that air pollutants, such as NO_2_, SO_2_, and O_3_, had a significantly positive correlation with higher mortality [3]. According to the 2017 China Ecological Environment Situation Report [4], 70.7% of the 338 prefecture-level cities in China failed to reach the new ambient air quality standard, where the content of PM2.5 and PM10 exceeded the normal index. Such a situation could not only greatly increase the risk of cardiovascular diseases and tuberculosis [5, 6], but also easily lead to forest fire [7] and the greenhouse effect, causing damages to the environment with the incensement of CO_2_ concentration in haze air.

The air quality in some public places is significantly reduced due to the high density of personnel flow and enclosed environment. Dust accumulates on the articles, resulting in serious health hazards [8]. People who spend too long in a confined environment with poor air quality are prone to dizziness, coughing, difficulty breathing [9, 10], and even DNA damage in oral and buccal mucosal cells [11]. For example, while providing a variety of indoor entertainment, Internet cafes have also become typical indoor air pollution sites, where the pollution is usually dominated by tobacco smoke. A survey in South Korea showed that smog pollution exists in 65% of domestic Internet cafes, and PM2.5 concentration is 2.7 times higher than the 24-hour exposure standard [12]. This means that while many people are active in various Internet cafes, the closed environment in the Internet cafe not only endangers the health of these people, but also has a negative impact on public environmental hygiene.

A deep understanding of air pollution depends on air pollution testing. Commonly used air quality monitoring solutions include dust image capture processing, assistive robot for air quality monitoring, and IAQ air detection framework [13–17]. Their characteristics are shown in Table 1. These methods are all dependent on air sensors to convert the collected air component information into readable digital signals, for example, to measure the components contained in them, such as formaldehyde and dust. However, the usual examination is sampling at a certain moment, and the conclusions drawn are limited by the exposure time to varying degrees.

In order to further reveal the laws of air pollution, this work started with investigating the pollution of Internet cafes, using an atomic force microscope (AFM) to observe the changes of surface topography of the contaminated glass slides with the exposure time, and the corresponding mechanisms of pollutant adhesion on glass surface were addressed. The chemical composition of surface pollutants was further analyzed by an X-ray photoelectron spectroscopy. In addition, different cleaning agents were employed for washing the contaminated glass surface, and the best cleaning condition was obtained by comparing the cleaning effects.

## 2. Experimental Materials and Methods

Since ordinary glass slides with a smooth surface are easy to obtain in the laboratory, they were chosen as the carrier of pollutants. The glass was cut into small pieces of about 1 × 1 cm^2^ and ultrasonically cleaned in ethanol and deionized water in turn for later use. A medium-sized Internet cafe is chosen in the city as an air pollution site, which is about 300 m^2^ in area and equipped with 120 desktop computers, and the average attendance rate reaches 60%. During the experiment, the number of people online was 75-85, and the air-conditioning temperature was set to 23°C. The relative humidity was recorded as 45-55%. We choose a computer in the center of the room, place the cleaned glass sheet (with clean paper underneath) on the desktop, and set the exposure time of the glass sheet to 8 time periods, namely, 10 min, 30 min, 1 h, 2 h, 4 h, 8 h, 16 h, and 24 h. At the end of each exposure time, the slide samples were put in the cleaning storage box with the tweezers and sealed for further tests. Do this in sequence until the end of 24 h and all samples are collected.

The AFM presents excellent three-dimensional imaging ability, making the image of pollutants clearer. An AFM (E-Sweep, Hitachi, Japan) was used to study glass surface topography and roughness changes in the present study. The nominal radius of the silicon nitride tip (MLCT, Veeco Instruments Inc, USA) is 20 nm, and the normal sprint of the cantilever is 0.1 N/m. An X-ray photoelectron spectroscopy (XPS; AXI Sultra DLD, Kratos Co. Shimadzu, Japan) was employed to analyze the chemical composition of the contaminated glass surface. There are three types of cleaning fluids prepared, i.e., 75% (v/v) alcohol (marked as #1 for simplicity), glass cleaning fluid (the main proportion is 10 wt.% C4 alkyl polyoxyethylene ether, 2 wt.% C12 sodium alkylbenzene sulfonate, 5% wt.% sodium toluene sulfonate, 3 wt.% ethanol, and 80 wt.% water) mixed with 6 wt.% NaHCO_3_ solution (the volume ratio of the two is 1 : 1; #2), and glass cleaning fluid (#3). The glass samples were ultrasonically cleaned in different cleaning fluids, and the exposed surfaces were put facing up [18]. The cleaning time is set as 1 min, 2 min, and 5 min. The AFM was also used to evaluate the cleaning effects by topography detection.

## 3. Results and Discussion

### 3.1. Evolution of Glass Surface Topography after Different Exposure Time

**Figure 1:**
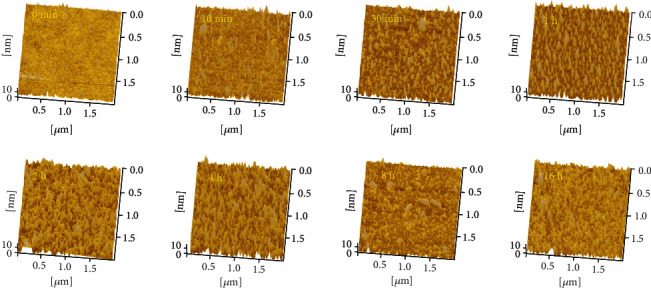
Topography of glass surface after different time exposure in the Internet cafe. Here, 0 min refers to the glass slide that has been cleaned but not exposed.

Figure 1 shows the morphological changes of glass surface after 0-24 h exposure in the Internet cafe. It can be seen that the exposure time has a significant impact on the detailed surface asperities of the slide. At 10 min, more microprotrusions can be observed on the initially smooth glass surface. As the exposure time increases to 30 min, protruding structure increases significantly. By 2 h, the raised structures are more obvious, like bamboo shoots after rain. It is shown to some extent that when pollutants in the air initially deposit or adhere to the glass surface, they tend to condense themselves first. As the exposure time continuously increases to 24 h, the surface morphology gradually becomes smooth, suggesting that the contaminant deposition tends to be stable (although its thickness will continue to increase). The entire contaminating process can be described as a gradual transition from discontinuous to uniform coverage.

**Figure 2:**
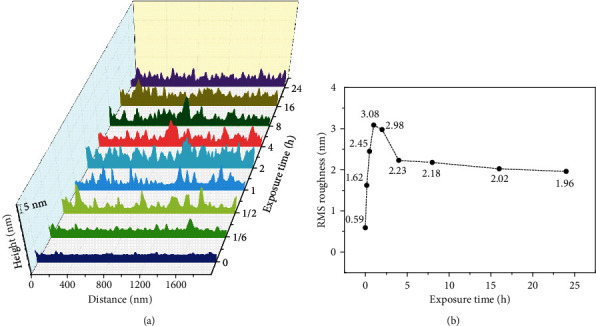
The evolution of (a) surface profiles and (b) RMS roughness value of glass slide with different exposure time in the Internet cafe. The surface profiles and roughness were obtained from Figure 1, and the former was only for demonstrating surface fluctuations.

**Figure 3:**
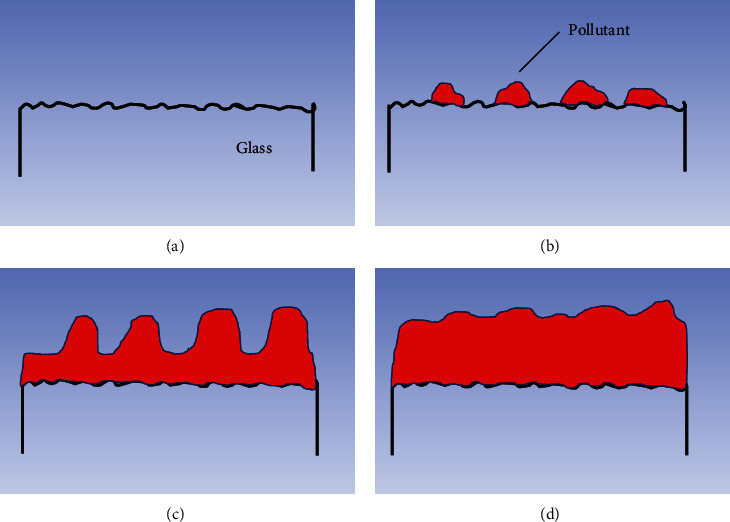
Schematics showing contaminant deposition on the glass surfaces in Internet cafes with exposure time. (a) Original glass surface, (b) initial aggregation of pollutant particles, (c) pollutant particle growth and interparticle deposition, and (d) full coverage.

Figure 2 compares the changes of surface profiles and roughness value of the slide glass. It can be seen from Figure 2(a) that the profiles change little within 10 minutes. Starting from 30 minutes, hillock-like structure appeared on the profile, further illustrating that the pollutants are characterized by aggregation and adsorption. When it comes to 24 h, the change of the profile tended to be gentle, indicating that the deposition process tended to be stable. Correspondingly, it can be seen from Figure 2(b) that RMS roughness value shows a trend of first rising and then falling, and the value at any time is higher than the value at 0 min (clean surface), which is attributed to the adsorption of air pollutants. The adsorption of air pollutants on the glass surface undergoes initial aggregation, particle growth and interparticle deposition, and final full coverage, which causes the roughness to rise and fall over time (Figure 3). In the ascending section, from 30 min to 1 h, the number of particles increased, and the particle diameter expanded from 0.05 *μ*m to 0.1 *μ*m. Pollutants were selectively aggregated and adsorbed, and the surface protrusions increase rapidly, causing an upward trend of the surface roughness value. Among them, the maximum height difference of particles is 3-4 nm in 10 min, 5-6 nm in 30 min, and 6-7 nm in 1 h, respectively, which is consistent with the large change of the profile from 30 min to 1 h in Figure 2(a). Pollutants tended to aggregate into microbumps and gradually became larger, leading to the highest roughness at about one hour, which accords with the phenomenon that the average height of the profile in Figure 2(a) reaches the highest. In the descending section, from 1 h to 4 h, interparticle deposition of pollutants became overwhelming, and more pollutant particles were adsorbed and filled between the valleys. Correspondingly, the height difference between the particle peaks and the valleys gradually decreased, and the surface roughness value decreased greatly, but the overall roughness value is still high. After that, from 4 h to 24 h, the interparticle deposition process slowed down, causing the peak-to-valley height difference to decrease from 6-7 nm to 3-4 nm, and the roughness value decreased more steadily.

### 3.2. Chemical Composition of Surface Pollutants

**Figure 4:**
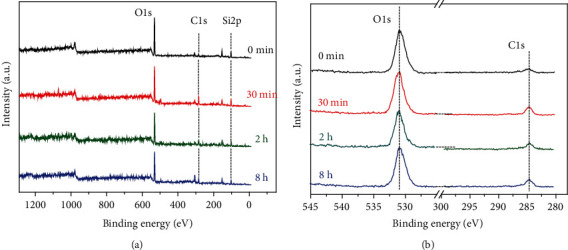
XPS spectrum of the surface after different exposure time. (a) Full spectrum and (b) O1s and C1s spectra. Here, 0 min refers to the glass slide that has been cleaned but not exposed.

In order to further study the chemical composition of surface contaminants, XPS detection was used to analyze the surface of the glass slide exposed for different periods. As shown in Figure 4(a), O1s, C1s, and Si2p signals are observed on the surface of the slide at 0 min (clean glass surface), 30 min, 2 h, and 8 h, respectively, where other peaks are from the glass substrate. Compared with the clean glass surface, it is noted that no new elements were found on the glass surface after the pollution, including sulfur dioxide and other sulfides usually contained in plastic packaging of Internet cafes. In Figure 4(b), C1s and O1s peaks, correspond to 284.8 eV and 531.08 eV, on the surface of the glass slide are not significantly different. The former corresponds to C-C and C-H bonds of hydrocarbons, and relatively higher intensity was detected on the contaminated glass surface than the clean surface. It is therefore deduced that the hydrocarbons mainly come from Internet cafe pollutants. In addition, for different types of bonds between C and O, i.e., C=O or C-O, the binding energy is generally greater than 284.8 eV; however, hardly any existence of these C peaks could be detected by XPS analysis. Considering that XPS analysis is carried out in a vacuum, gaseous CO_2_ has been desorbed from the surface. In other words, the adsorption ability of CO_2_ is weaker than that of pollutants. Therefore, the pollutants of Internet cafes are mainly hydrocarbons, and the chemical composition of air pollutants has not changed over time.

In addition, a quantitative analysis of the changes in the peak signals of O1s and C1s over time was carried out. As shown in Table 2, the atomic ratio of O to C on the glass surface decreases with the exposure time. It is noted that the O content ratio in the pollutants can be lower than that in the glass matrix, which further supports the inference that Internet cafe pollutants are mainly hydrocarbons.

### 3.3. Surface Cleaning

**Figure 5:**
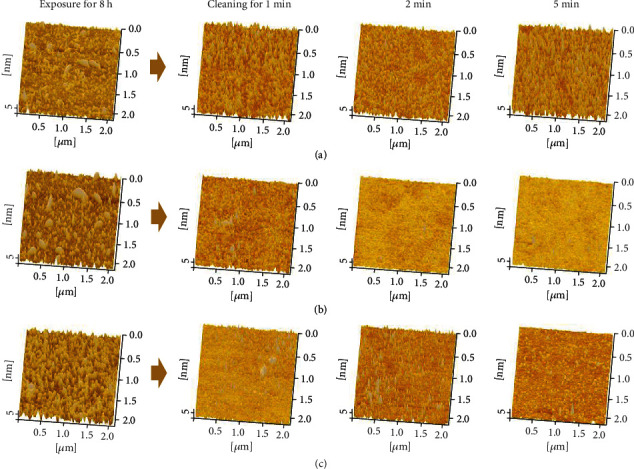
Surface topography of the slide after being cleaned with cleaning solutions for different time (1, 2, and 5 min). (a–c) The surface topography of the slide cleaned by agents #1, #2, and #3, respectively.

**Figure 6:**
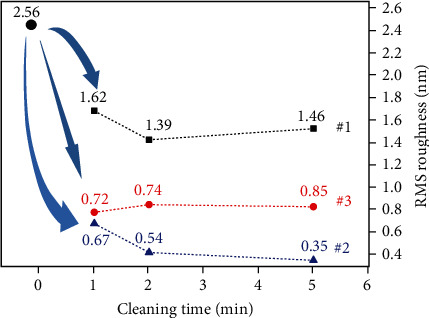
Changes of RMS roughness of the glass slide exposed for 8 h after being cleaned with different agents (#1-#3) for 0-5 min. Here, 2.56 nm shows the roughness of the glass surface exposed for 8 h.

In Figure 5, after cleaning with three different cleaning solutions, the adhesion of contaminants on the surface of the glass slide is reduced, the protruding parts on surface morphology are significantly reduced compared with the original surface at 8 h, and the peaks of the surface profile become lower. To further investigate the cleaning effect, RMS roughness of the cleaned surface was further compared. Among them, surface roughness treated by cleaning agent #1 falls less than the others, which means agent #1 has the relatively worst cleaning effect. Correspondingly, the surface roughness in Figure 6 decreases obviously after the cleaning with agent #2. The longer the cleaning time, the better the cleaning effect. When the cleaning time reaches 5 min, the RMS roughness treated with agent #2 (0.35 nm) comes to the bottom. In contrast, the cleaned surface does not change much during this period for agent #3, and the cleaned surface still has obvious microconvex peaks (Figure 5(c)). Through the longitudinal comparison of the surface topography of the glass slide, the surface of the glass slide after cleaned by agent #2 is smoother, with significantly fewer peaks, and the cleaning effect is much better than that of agents #1 and #3.

The particle size of air dust in Internet cafes is small (Figure 1), and it is easy to be adsorbed on solid surfaces, especially the display surface with a higher electrostatic voltage on the back. Pollutants entrain degradable acid radicals, showing weak acidity [19]. Based on XPS detection, it is pointed out that pollutants in Internet cafes are mainly C-H compounds, and some will adhere to the glass slide in the form of grease [20]. The NaHCO_3_ in agent #2, as a strong soluble base and weak acid salt, is hydrolyzed to alkaline (HCO_3_^−^ + H_2_O = H_2_CO_3_ + OH^−^). Acid-base neutralization reaction and alkaline environment of NaHCO_3_ solution make the attached little grease undergo saponification reaction and hydrolysis [21], achieving better decontamination effect and reduce surface roughness.

Through this study, the surface topography of exposed glass slide was studied by an AFM, and it is noted that the pollutant adsorption phenomenon can experience various stages. However, the detailed mechanism for the pollutants deposition changing from initial aggregation to interpillar deposition still needs further investigation. In addition, considering AFM has been used for force spectrum measurement and dynamic force measurement [22, 23] of interaction strength in biological pharmacology and metal materials, and further study can also start from the perspective of adhesion behaviors. Investigate adhesion force between air pollutants and the force between air pollutants and clean surface to find out more information for interpreting air pollution.

## 4. Conclusions

This paper started from the pollution of Internet cafes, focusing on the changes in surface morphology of the contaminated glass slides with exposure time. With X-ray photoelectron spectroscopy, chemical element composition and elemental state of the pollutants were further analyzed. In addition, the cleaning of the glass surface was investigated to reobtain a clean surface. The main conclusions obtained are as follows.

1. With the increase of exposure time of the glass sheet to the air, the deposition process of air pollutants on the surface of the long-standing glass slide approaches a transition process from discontinuous deposition to uniform coverage, which also directly leads to the overall trend of RMS roughness value first rising and then falling over time
2. The surface of the glass slide after being placed for a long time only has the accumulation of pollutants on the physical level, and there is no new generation of compounds on the chemical level. Pollutants are mainly hydrocarbons, and no C=O or C-O bonds in pollutants are not observed from XPS detection
3. Cleaning tests with different cleaning agents show that NaHCO_3_-contained solution can be the most effective one with saponification reaction and hydrolysis to remove the adsorbed contaminations

## Figures and Tables

**Table 1:** Common air quality research programs and their characteristics [13–17].

Detection schemes	Operation descriptions
Dust image capture and processing	Locating accurately dust source and providing effective countermeasures
Real-time wireless sensor networks	Low power and real-time visualization
Assistive robot for indoor air quality monitoring	Expansibility and information sharing
IAQ air detection framework	Future operation and maintenance of the pilot area and long-term significance
Semiconductor gas sensor networks	Simple information, low accuracy, and low cost

**Table 2:** Atomic ratio of O to C of the glass surface with the exposure time.

Exposure time	Atomic ratio
0 min	5.21
30 min	2.41
2 h	1.94
8 h	1.66

## Data Availability

The data used to support the findings of this study are included within the article.
